# A Scoping Review of Psychological Sense of Community among Community-Dwelling Older Adults

**DOI:** 10.3390/ijerph19148395

**Published:** 2022-07-09

**Authors:** Thomas D. Buckley

**Affiliations:** Department of Psychiatry, School of Social Work, University of Pittsburgh, Pittsburgh, PA 15260, USA; tdb47@pitt.edu

**Keywords:** psychological sense of community, older adults, age-friendly, ecological theory of aging, neighborhoods

## Abstract

Psychological sense of community (PSOC) is an important construct for health and well-being outcomes for community-dwelling older adults. Drawing on the Ecological Theory of Aging and the Age-Friendly Cities (AFC) framework, this scoping review explored how PSOC has been used in research with community-dwelling older adults. This study examined antecedents, correlates, and outcomes of PSOC, with a focus on relevance to theory and practice. Databases were searched between 1986 and 2021 for peer-reviewed journal articles. Searches identified 582 unique articles, and 28 were included in the final sample. Three primary themes emerged in the synthesis: relevance to the AFC framework, PSOC as a predictor of health and well-being outcomes, and the role of PSOC in relocation. Findings from this review show that PSOC serves as a mechanism that links the social and physical AFC environments with health and well-being outcomes. This review also presents mechanisms for how features of the environment relate to PSOC. These findings demonstrate the role of PSOC as a resource to improve person–environment fit. Results from this review can be used to guide future research and inform theory, policy, and practice.

## 1. Introduction

Psychological sense of community (PSOC) describes an individual’s feeling that they are part of a social structure that is supportive, present, and dependable and is characterized by interdependence, mutual responsibility, and a collective consciousness [1]. PSOC serves as an important concept for understanding the interaction between community-dwelling older adults and their environment. For example, PSOC is related to aspects of the environment or neighborhood such as the built environment [2], neighborhood characteristics such as public space and population density [3], and perceptions of one’s environment [4]. Research with older adults shows that PSOC predicts lower levels of depressive symptoms and better health [5,6], as well as better life satisfaction [7,8]. In a study of older adult earthquake survivors, PSOC served as a protective factor for mental health [9]. PSOC also acts as a resource to respond to environmental demands and psychological needs, leading to better well-being outcomes [10].

This emerging field of research underscores the relationship of PSOC to well-being outcomes and shows the influence of the physical and social environment on creating PSOC. However, current research has yet to summarize these relationships and place them within existing theoretical frameworks. Bess et al. [11] argue that PSOC, as a construct, is simultaneously easily understood yet difficult to agree on specific mechanisms on why or how it leads to better outcomes across different demographic groups. Therefore, it is vital to the fields of gerontology and community psychology to gain a deeper understanding of how PSOC has been researched with older adults and to explore why relationships occur. The aim of this study was to conduct a scoping review to explore how PSOC has been studied with community-dwelling older adults. Scoping reviews use a strong methodological framework to map and synthesize evidence around a specific topic to inform practice, policy, and future research [12,13].

McMillan and Chavis [14] further defined PSOC as a “feeling that members have of belonging, a feeling that members matter to each other, and a shared faith that member’s needs will be met through their commitment together” (p. 9). They identified four key domains and processes of PSOC: (1) *Membership*—a feeling of belonging or shared personal relations; (2) *Influence*—a sense of mattering and making a difference in one’s community, and the bidirectional relationships between members and their communities; (3) *Fulfillment of needs*—the idea that a member’s needs will be met by the resources provided through being in the community; and (4) *Shared emotional connection*—a commitment and belief that members have shared and will continue to share a history, common places, time with each other, and events or experiences. These four domains work together to create and sustain PSOC. This scoping review references the four domains of PSOC (membership, influence, fulfillment of needs, and shared emotional connection) to describe mechanisms of how PSOC operates for community-dwelling older adults.

This review uses the Ecological Theory of Aging [15] and the World Health Organization’s (WHO) Age-friendly Cities framework (AFC) [16] to demonstrate the relevance of PSOC for community-dwelling older adults. The relationship between an older adult and their community is essential to environmental gerontology. Lawton and Nahemow’s [15] Ecological Theory of Aging seeks to understand interactions and assess quality of fit between older adults and their physical and social environment. Good person and environment fit (P–E fit) leads to positive outcomes, whereas poor P–E fit leads to negative outcomes. Goodness of fit is best understood by evaluating the combination and interaction of personal characteristics and environmental influences. Person-related characteristics, referred to as *personal competence*, include sociodemographic characteristics, health, functioning, and social resources. The environment around an individual creates demands or strains, also referred to as *environmental press*. Older adults with higher levels of personal competence are better able to respond to and meet the needs or stress imposed on them by their environment [15]. Conversely, older adults with lower levels of competence function best in environments with lower demands. Poor outcomes result when an older adult’s level of competence is insufficient to meet the demands of environmental press. PSOC can be conceptualized as a resource one can draw on to respond to environmental press, and conversely, absence of PSOC may result in poor P–E fit.

Policy initiatives emphasize the importance of P–E fit and the role of community factors in promoting the well-being of older adults. In 2005, the World Health Organization (WHO) conceived the Age-Friendly Cities (AFC) framework to respond to global population aging trends and to promote living environments that enhance health and well-being of older adults [16]. AFC consists of eight general domains: *Housing*, *Social Participation*, *Respect and Social Inclusion, Civic Participation and Employment, Communication and Information, Community Support and Health Services, Outdoor Spaces and Buildings*, and *Transportation* (for more detail on each domain, see [16]). This framework can be used by local governments and policy makers to aid in designing the environment to be more conducive to the needs of and opportunities for older adults.

The emergence of AFC demonstrates a shift in research and practice from focusing on the individual level to the community level [17]. Thus, older adults’ perceptions of their communities become an essential focus for research. A primary goal of the AFC framework is to design communities and environments that meet the needs of older adults, and as Nowell and Boyd [10] point out, PSOC is produced when the community can meet residents’ needs. While PSOC is not part of the AFC framework, it is an important construct for understanding how environmental influences and community-level factors affect health and well-being outcomes. In fact, PSOC acts as a mediator to link age-friendly environment features with life satisfaction [7], subjective well-being [4], and self-rated health [6].

Using scoping review methodology, this paper will explore how PSOC has been used in research with community-dwelling older adults and place findings in the context of the AFC framework and the Ecological Theory of Aging. Specifically, this study examines antecedents, correlates, and outcomes of PSOC, with a particular focus on understanding the mechanisms of PSOC for community-dwelling older adults and the relevance to theory and practice.

## 2. Materials and Methods

This review followed the framework for scoping reviews presented by Arksey and O’Malley [12] and refined by Levac et al. [13]. The process consists of five stages: (1) identify the research question, (2) identify relevant articles, (3) select studies, (4) chart data, and (5) collate, summarize, and report the results. Results are reported in accordance with the PRISMA extension for scoping reviews [18] (Supplemental Table S1). Quality appraisals of articles did not take place, as this is not undertaken in scoping reviews [12,13].

The research questions guiding this scoping review are: “How has the construct of PSOC been used in research with community dwelling older adults?” and “How do the Ecological Theory of Aging and the AFC framework help explain the role of PSOC for community dwelling older adults?”

### 2.1. Identifying and Selecting Articles

Three databases (EBSCO, Web of Science, and ProQuest) were searched with a date range of 1986 (the year of McMillan and Chavis’ seminal work on PSOC) to March 2021. Searches in each database specified: search entire database, include only peer-reviewed journals, English full text available (when possible), and date range of 1986–March 2021. The following search terms were used in each database: (“older adult *” or elder * or geriatric * or aging or senior * or “older people” or “aged 60” or 60+ or “aged 65” or 65+) AND “sense of community”. Other articles were identified through chain citation searching.

Eligibility criteria were established prior to searching, and additional criteria were developed *post hoc* as familiarity with the literature increased [12]. Eligibility criteria were:

- Only peer-reviewed, empirical articles were included (i.e., quantitative, qualitative, mixed-methods, case studies, and review articles). Non-empirical (e.g., book reviews, letters to the editor) or non-peer-reviewed publications (e.g., theses and dissertations) were excluded.
- Articles were excluded that did not have full text available in English.
- Articles were excluded if the sample was not older adults or if there was a mixed age sample.
- Articles with samples from nursing homes, assisted living facilities, retirement homes, cohousing, or other older-adult-specific housing were excluded, as this review focused on community-dwelling older adults.
- Articles were excluded that used the phrase “sense of community” in ways not related to PSOC. While the literature on PSOC uses “sense of community” or “psychological sense of community” interchangeably, some literature outside of PSOC uses the general phrase “sense of community” to refer to unrelated constructs, such as a general reference to perceptions of community but with no reference at all to the construct of PSOC.
- Articles were excluded if PSOC was a minor focus, such as only making minor references to PSOC in the introduction or conclusion or using it as a minor variable in analyses as opposed to a key construct in the hypothesis or model.

### 2.2. Data Charting

Charting involves synthesizing and interpreting data based on key items of information from the studies reviewed [12]. Data were extracted from selected articles based on general study characteristics (author(s), year of publication, journal, aim/goal/purpose, research design, sample characteristics, and statistical or analytical approach) and on specific PSOC topics, such as hypotheses, conceptualization of PSOC, type of variable PSOC was in analysis, constructs or variables tested with PSOC, and main results. Data were also charted on how articles fit into research themes and their relevance to the Ecological Theory of Aging or AFC.

### 2.3. Collating, Summarizing, and Reporting the Results

This stage provides an overview of all material reviewed and presents key themes [12]. Information was first presented on the article selection process and general study characteristics. Then, articles were grouped into three themes based on research topics and constructs tested with PSOC. The three themes were: relevance to the AFC framework, PSOC as a predictor of health and well-being outcomes, and the role of PSOC in relocation. Some articles were grouped in multiple themes. These themes emerged as studies were reviewed and data extracted. This involved an iterative process of developing themes, re-appraising articles, taking detailed notes, and revisiting the data-charting stage. Research trends and findings were then framed vis-à-vis AFC and the Ecological Theory of Aging.

A crucial step in scoping reviews is to ensure consistency among reviewers in the process, such as article selection and data charting [13], as well as in analyzing how and why articles fit into each theme. Each step in this review was primarily completed by a sole researcher. To help reduce bias, a detailed journal was kept, noting decisions and rationale at each step in the process, to improve consistency in judgments. Additionally, librarians and topical area experts in gerontology and social work were consulted throughout the process. The librarian aided in the identifying and selecting articles stages, including assisting in developing the search strategy and establishing eligibility criteria. Topical experts also assisted the author in establishing eligibility criteria and developing themes, as well as helping resolve ambiguity among articles during the selecting studies and collating, summarizing, and reporting stages.

## 3. Results

**Figure 1:**
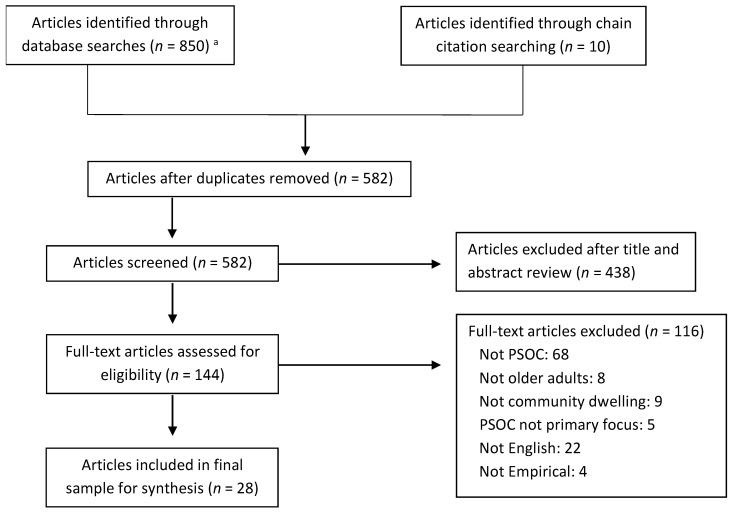
PRISMA Diagram for Search and Selection Process. *Note*. PRISMA = Preferred Reporting Items for Systematic Reviews, PSOC = psychological sense of community. ^a^ Databases searched (*n* for each): EBSCO (*n* = 398), Web of Science (*n* = 283), ProQuest (*n* = 161).

Figure 1 shows the PRISMA diagram for the scoping review. Initial searches yielded 860 articles (EBSCO = 398, Web of Science = 283, ProQuest = 161, and other sources = 10). After removing duplicates, 582 unique articles were reviewed. First, titles and abstracts were screened based on the eligibility criteria, and 438 articles were excluded. Then, the remaining full text of 144 articles were further reviewed, and 116 articles were excluded. The final sample included 28 articles.

### 3.1. Study Characteristics

Table 1 presents information on the final sample of 28 articles, including general study characteristics and information on PSOC for each article. Articles were published between 2003 and 2021. A total of 24 studies used quantitative analyses, and 4 used qualitative methods. Only one was longitudinal [19]. In statistical analyses, PSOC was most used as a mediator (*n* = 12), followed by independent variable (*n* = 10), dependent variable (*n* = 5), and moderator (*n* = 2). Some articles used PSOC in multiple ways, such as a mediator and moderator [20] or as independent variable and moderator [9]. All four qualitative studies operationalized PSOC as a key construct to guide or frame their analysis and results.

Studies mostly used age ranges of 60 years or older to define “older adults”, while a smaller number used cutoffs of 55+ (*n* = 4) or 50+ (*n* = 4), some citing WHO [21] guidelines for age 50 and older in developing countries representing “old age.” Fifteen studies were set in Hong Kong or China, eight in the USA or Canada, three in Australia or New Zealand, and one each in Italy and South Africa. Of the USA samples, five were from the Population Study of Chinese Elderly in Chicago (PINE), and one study in New Zealand was with Chinese immigrants [22]. In total, 21 of 28 studies contained Chinese older adults; the remaining were samples of White older adults (*n* = 6), and another was with older women in South Africa [23].

Four articles conceptualized PSOC as part of a latent construct: “environmental factors” [24], “neighborhood factors” [19], “resource variable” [9], or “social inclusion” [25]. The term “community” traditionally refers to a geographic or physical community, but it may also include relational communities that transcend physical and spatial location [11]. Most studies used “community” in geographical contexts, such as the neighborhood or local area. However, this was often not explicitly stated. Only the qualitative studies specifically defined community beyond geographic features. These communities were: exercise groups, sports competitors, communities of emigration, and among older women who were forcibly relocated during apartheid in South Africa.

### 3.2. Research Topics in Synthesis

Articles were grouped into three themes based on the topic in which PSOC was studied: (1) WHO AFC framework; (2) PSOC, health, and well-being; and (3) relocation.

#### 3.2.1. WHO AFC Framework

As described earlier, the WHO [16] AFC framework contains eight domains: *Housing, Social Participation, Respect and Social Inclusion, Civic Participation and Employment, Communication and Information, Community Support and Health Services, Outdoor Spaces and Buildings, and Transportation.* In total, 20 articles were grouped into this theme. Each article evaluated PSOC as an outcome of AFC and/or as a mediator between AFC and well-being or health outcomes. These articles either directly referenced the AFC framework, or the main topics covered were clearly related to specific domains. For example, if an article explored housing or outdoor spaces but did not specifically reference AFC, the article was grouped into the AFC theme within those domains.

AFC is often divided into the physical environment (i.e., *Housing, Community Support and Health Services, Outdoor Spaces and Buildings, and Transportation*) and the social environment (i.e., *Social Participation, Respect and Social Inclusion, Civic Participation and Employment, and Communication and Information*). Articles were further grouped into these categories, with some found in both.

**Physical Environment.** Nine articles fell within the physical environment category of the AFC framework, which includes the domains of *Outdoor Spaces and Buildings*, *Community Support and Health Services*, *Transportation*, and *Housing*.

*Outdoor Spaces and Buildings* touches on topics such as pleasant and clean environments, green spaces, places for rest and leisure, age-friendly pavements, safe pedestrian crossings, age-friendly buildings, and secure or safe environments (WHO, 2007). Multiple studies found this domain to be related to better PSOC [2,6,7,26].

Several studies further examined public and outdoor spaces and found mixed results with PSOC. On one hand, Zhang et al. [3] found a positive association between public spaces and PSOC. Likewise, Toohey et al. [27] suggested that neighborhoods with more walkability and parks may support better PSOC among older adults. However, in a study by Guo et al. [2], more green space was associated with less PSOC. They speculate that, in their sample, areas with more green space and vegetation may be more socially disconnected, thus resulting in lower PSOC. While these studies mostly focused on *quantity* of space, the *quality* of open spaces, such as parks, may be a better predictor of PSOC [28].

The goal of age-friendly *Community Support and Health Services* is to maintain older adults’ health and independence. This domain includes topics such as accessibility of care, range of services, home care, services to promote active aging, residential facilities, and networks of community services [16]. Zhang et al. [3] and Guo et al. [2] used objective methods to measure the availability of senior services and the number of health services, respectively, both of which predicted better PSOC. Other studies used subjective measures, such as perceptions of availability of or satisfaction with such services, and found that these also resulted in better PSOC [6,7].

*Transportation* refers to accessible and affordable public transportation and age-friendly driving conditions, both of which can promote social and civic participation and access to community or health services [16]. Greater satisfaction with transportation [25] or better perceptions of transportation [6] were associated with better PSOC. *Housing* touches on structure, design, location, and choice, with focuses on age-friendly options, ability to age in place, proximity to key resources, and safety [16]. This was only represented in one study, where it was positively associated with PSOC [6]. Last, Zhang and Zhang [4] used a general measure of perceptions of the physical environment, which was a predictor of better PSOC.

**Social Environment.** Thirteen articles fell within the social environment, which includes the AFC domains of Social Participation, Civic Participation and Employment, Communication and Information, and Respect and Social Inclusion.

*Social Participation* refers to the need to increase the accessibility and affordability of social activities and other strategies to prevent social isolation [16]. The relationship of social participation and PSOC was mixed in this scoping review. Using data from the PINE study, Lai et al. [24] grouped PSOC with other constructs under “environmental factors” and found that it predicted more social engagement. Other studies explored the reverse association of these constructs, looking at how social factors predict PSOC. In a different study using the PINE data, engagement in social activities and increased social support were associated with more PSOC [29]. Likewise, more community involvement was associated with higher PSOC among older women in China [30]. Au et al. [7] and Yu et al. [6] specifically used *Social Participation* as a variable in their analyses and found that it predicted better PSOC.

Leisure activities also fall under *Social Participation* in the AFC framework, as they are thought to be an effective strategy for increasing social connections and reducing social isolation [16]. Two qualitative articles set in Australia explored PSOC with older adults participating in a sports contest [31] and an exercise group [32]. These studies suggested that participating in these activities presents an avenue to build PSOC and feel part of multiple communities ranging from the micro level (community with other participants) to the macro level (local communities and larger community). In another study, Toohey et al. [27] found that frequent dog walking was a predictor of higher PSOC.

Three articles were related to *Civic Participation*, which involves encouraging active roles for older adults in civic processes, such as elections or community improvement, and increasing access to employment and volunteer activities [16]. Okun and Michel [33] found that, among older adults in the United States, PSOC predicted more involvement in volunteering. Likewise, PSOC was associated with better psychological well-being among older adults who volunteer [34]. For older adults in rural areas in the USA, higher PSOC was associated with more involvement in community improvement activities [35].

*Communication and Information* is thought to promote social connection and improve access to information and services [16]. Two studies explored this AFC domain, with mixed results. Yu et al. [6] found that the domain was associated with better PSOC. However, Fang et al. [36] found that using more information and communication technology, such as computers or smartphones, was associated with lower PSOC, particularly for lonelier older adults.

*Respect and Social Inclusion* speaks, in part, about promoting intergenerational programming, economic inclusivity, and addressing ageism [16]. One study found *Respect and Social Inclusion* was positively associated with PSOC [6]. Yao et al. [37] reported a chain mediation from perceived age discrimination to life satisfaction through national identity and PSOC, showing that PSOC perhaps protects against discrimination.

**PSOC as a Mediator.** Eleven articles in the AFC theme used PSOC as a mediator (see Table 1). In each of these studies, AFC characteristics were independent variables, and measures of well-being or health were outcome variables. For example, PSOC mediated the relationship between *Social Participation* and *Community Support and Health Services* with life satisfaction [7], the physical and social environment of AFC with health [6], perceived residential environment with subjective well-being [4], and neighborhood characteristics with well-being [3].

#### 3.2.2. PSOC, Health, and Well-Being

The second theme was the relationship between PSOC and health or well-being. Counting those found in other themes, 17 articles in total were grouped into this theme.

Multiple studies examined life satisfaction or well-being as outcomes. PSOC was routinely a significant predictor of better life satisfaction [8,37,38] and well-being [2,3,30,34]. Two other studies found PSOC was a mediator between AFC domains and life satisfaction [7] and between perceived residential environment and well-being [4]. Several of these studies further explored the role of resilience. Resilience was a moderator between PSOC with life satisfaction [8] and well-being [30] and was a significant mediator between PSOC and life satisfaction [38].

**Table 1:** Data Extraction and Charting for Articles in Final Sample.

Author (Year) [Citation]	Sample Characteristics	Research Design and Use of PSOC in Analysis	Main Findings Related to PSOC	Research Topics in Synthesis
Au et al. (2020) [7]	Hong Kong, 55+ (*N* = 898)	Quantitative. PSOC as mediator	AFC domains of social participation, community/health services, civic participation and employment, and outdoor spaces and buildings were associated with higher PSOC. PSOC mediated relationships of social participation and community/health services with life satisfaction.	AFC Framework (Physical and Social); PSOC, Health, and Well-being
Dionigi and Lyons (2010) [32] ^c^	Australia (white exercise group participants), 65+ (*N* = 10)	Qualitative. PSOC guided analysis	Participating in exercise group increased PSOC. Participants felt PSOC in different levels from micro to macro and among different types of communities.	AFC Framework (Social)
Dong et al. (2014) [39] ^a^	USA (Chinese immigrants), 60+ (*N* = 3159)	Quantitative. PSOC as DV	Participants reported high PSOC. Correlates of PSOC included higher income, more children, longer length living in the U.S., longer length residence in their community, and better health and quality of life.	PSOC, Health, and Well-being; Relocation
Fang et al. (2019) [36]	Hong Kong, 60+ (*N* = 738)	Quantitative. PSOC as DV	Information and communication technology use was associated with lower PSOC among older adults who had higher loneliness.	AFC Framework (Social)
Guo et al. (2021) [2]	Hong Kong, 60+ (*N* = 1553)	Quantitative. PSOC as mediator	Results showed pathways from objective built environment to mental health and subjective well-being outcomes through paths of perceived built environment and PSOC. PSOC was positively associated with perceived built environment, mental health, and subjective well-being. More green space associated with lower PSOC; more health services associated with higher PSOC.	AFC Framework (Physical); PSOC, Health, and Well-being
He et al. (2020) [25]	Hong Kong, ~60+ (*N* = 271)	Quantitative. PSOC as IV and mediator	PSOC was part of latent variable, social inclusion. This predicted better psychological well-being but not physical well-being. Social inclusion mediated relationship between satisfaction with transportation and psychological well-being. Social inclusion and satisfaction with transportation were positively correlated.	AFC Framework (Physical); PSOC, Health, and Well-being
Lai et al. (2019) [24] ^a^	Chicago (Chinese immigrants), 60+ (*N* = 3159)	Quantitative. PSOC as IV	PSOC associated with more engagement in social activities but not with cognitive activity engagement.	AFC Framework (Social); Relocation
Lai et al. (2021) [20]	Hong Kong, 60+ (*N* = 1793)	Quantitative. PSOC as mediator and moderator	PSOC moderated relationship between education and health; those with higher PSOC had stronger association between lower education and worse health. PSOC mediated relationship between disposable income and health; less disposable income predicted lower PSOC and worse health.	PSOC, Health, and Well-being
Li et al. (2014) [22] ^c^	New Zealand (Chinese immigrants), 60+ (*N* = 32)	Qualitative. PSOC guided analysis	Participants felt PSOC in their current local community as well as their former home community. Strategies and barriers for building PSOC in both these settings are discussed.	Relocation
Li et al. (2011) [9]	China (survivors of an earthquake), 55+ (*N* = 298)	Quantitative. PSOC as IV and moderator	PSOC moderated relationship between earthquake-related distress and depressive symptoms. PSOC associated with fewer depressive symptoms.	PSOC, Health, and Well-being
Liu and Besser (2003) [35]	USA (white, rural areas), 65+ (*N* = 2802)	Quantitative. PSOC as IV	PSOC predicted higher level of involvement in community improvement activities.	AFC Framework (Social)
Lyons and Dionigi (2007) [31] ^c^	Australia (white participants in sports competition), 55+ (*N* = 110)	Qualitative. PSOC guided analysis	Four themes emerged related to PSOC and four domains from McMillan and Chavis (1986): shared sporting interest (membership), comrades in continued activity (emotional connection), relevant life purpose (needs fulfillment), and giving back (influence). PSOC lasts beyond the episodic event of a sports competition.	AFC Framework (Social)
Okun and Michel (2006) [33]	USA (white), 60+ (*N* = 653)	Quantitative. PSOC as IV	PSOC predicted increased likelihood of volunteering.	AFC Framework (Social)
Pozzi et al. (2014) [34]	Italy (white volunteers), 60+ (*N* = 143)	Quantitative. PSOC as mediator	Religiousness and sense of responsibility associated with PSOC, and PSOC predicts volunteer motivation and generativity. PSOC was mediator with outcomes of psychological well-being.	AFC Framework (Social); PSOC, Health, and Well-being
Roos et al. (2014) [23] ^c^	South Africa (females who were forcibly relocated during apartheid), 70+ (*N* = 11)	Qualitative. PSOC guided analysis	Sense of current and former communities were explored. Connections to place and sense of belonging emerged as themes, and personal history and context helped shape conceptions of community. There was stronger connection among peers of the same age group and those who shared common interests and beliefs than among generational groups and with current or past physical places.	Relocation
Tang et al. (2017) [29] ^a^	USA (Chinese immigrants), 60+ (*N* = 3159)	Quantitative. PSOC as DV	Social activity engagement and positive social support associated with higher PSOC.	AFC Framework (Social); Relocation
Tang et al. (2018) [5] ^a^	USA (Chinese immigrants), 60+ (*N* = 3159)	Quantitative. PSOC as IV	Higher PSOC associated with lower likelihood of reporting worse health and more depressive symptoms.	PSOC, Health, and Well-being; Relocation
Tang et al. (2020) [19] ^a,b^	USA (Chinese immigrants), 60+ (*N* = 2713)	Quantitative. PSOC as IV	PSOC not significantly associated with better cognitive functioning.	PSOC, Health, and Well-being; Relocation
Tang et al. (2021) [26]	Hong Kong, 50+ (*N* = 2247)	Quantitative. PSOC as mediator	PSOC mediated relationship between age-friendliness of built environment (outdoor spaces and buildings) with physical and mental health-related quality of life. Built environment positively associated with PSOC, and PSOC positively associated with quality-of-life measures.	AFC Framework (Physical); PSOC, Health, and Well-being
Toohey et al. (2013) [27]	Canada (white), 50+ (*N* = 884)	Quantitative. PSOC as mediator	PSOC does not mediate relationship between dog walking or neighborhood characteristics and recreational walking. Frequent dog walking associated with higher PSOC, and neighborhoods with lower education associated with lower PSOC.	AFC Framework (Physical and Social)
Yao et al. (2018) [37]	China, 60+ (*N* = 391)	Quantitative. PSOC as mediator	Results show chain mediation from perceived discrimination to life satisfaction through national identity and PSOC. PSOC positively associated with life satisfaction.	AFC Framework (Social); PSOC, Health, and Well-being
Yu (2021) [28]	Hong Kong, 55+ (*N* = 257)	Quantitative. PSOC as DV	Quality of space in one’s neighborhood environment associated with higher PSOC, but quantity of open space was not.	AFC Framework (Physical)
Yu et al. (2019) [6]	Hong Kong, 60+ (*N* = 1798)	Quantitative. PSOC as IV, DV, and mediator	PSOC mediated relationship between physical and social neighborhood environments and self-rated health. Overall, AFC and each AFC domain were positively associated with PSOC. Overall, PSOC and domains of needs fulfillment, influence, and emotional connection were positively associated with health.	AFC Framework (Physical and Social); PSOC, Health, and Well-being
Zhang et al. (2017) [8]	China (partnered couples), 60+ (*N* = 516)	Quantitative. PSOC as IV	PSOC associated with better life satisfaction. This was moderated by psychological resilience; those with lower resilience had weaker relationship of PSOC and life satisfaction.	PSOC, Health, and Well-being
Zhang et al. (2018) [3]	China, 60+ (*N* = 628)	Quantitative. PSOC as mediator	PSOC mediated relationship between neighborhood characteristics (i.e., public space, older adult population density, and senior services) and well-being. Neighborhood characteristic variables predicted higher PSOC, which was then associated with better well-being.	AFC Framework (Physical); PSOC, Health, and Well-being
Zhang (2019) [30]	China (females, moved from rural to urban), 50+ (*N* = 205)	Quantitative. PSOC as mediator	PSOC mediated relationship between community participation and relocation adjustment outcomes (depression, loneliness, well-being). PSOC directly associated with better relocation adjustments outcomes. Resilience moderated relationship of PSOC and relocation adjustment outcomes.	AFC Framework (Social); PSOC, Health, and Well-being; Relocation
Zhang and Zhang (2017) [4]	China, 50+ (*N* = 720)	Quantitative. PSOC as mediator	PSOC mediated relationship between perceived residential environment and measures of subjective well-being (satisfaction with life, meaning in life, and positive affect). Perceived residential environment was significant predictor of PSOC.	AFC Framework (Physical); PSOC, Health, and Well-being
Zheng et al. (2020) [38]	China, 60+ (*N* = 418)	Quantitative. PSOC as IV	PSOC associated with better life satisfaction, and this was mediated by psychological resilience.	PSOC, Health, and Well-being

*Note*. *N* = 28 articles. PSOC = Psychological sense of community, AFC = Age-Friendly Cities, DV = Dependent Variable, IV = Independent Variable. White = majority White sample. ^a^ Used data from Population Study of Chinese Elderly in Chicago (PINE). ^b^ Longitudinal study, all others are cross-sectional. ^c^ Defined “community” beyond geographic community or local neighborhood.

A number of articles explored topics of mental and physical health. PSOC was associated with less depressive symptoms in three studies: older adults following an earthquake [9], Chinese immigrants in the USA [5], and relocatees from rural to urban areas in China [30]. Two other studies showed that PSOC was associated with better overall mental health [2] and mental health quality of life [26].

While there was a clear link between PSOC and better mental health, there was less consensus with physical health. On one hand, several studies support the significant association between PSOC and physical health. Tang et al. [5] and Yu et al. [6] found that PSOC predicted better self-rated health, and Dong et al. [39] showed that PSOC was positively correlated with health. Similarly, PSOC was associated with better physical health-related quality of life [26]. However, a small group of studies found contrary results. Tang et al. [19] found no significant association between neighborhood characteristics (which included PSOC along with social cohesion and neighborhood disorder) and cognitive functioning. He et al. [25] included PSOC as part of a latent variable of “social inclusion” and found that this was associated with better psychological well-being but not physical well-being.

Other articles highlighted the role of PSOC as a protective factor for health and well-being. In a study with older adults in Hong Kong, PSOC moderated the relationship between education and self-rated health and mediated the relationship between income and self-rated health such that higher income predicted higher PSOC and, in turn, better self-rated health [20]. With respect to traumatic events, PSOC was found to moderate the relationship between exposure to an earthquake in China and depressive symptoms [9]. In this study, older adults with higher PSOC had fewer depressive symptoms even when they had more exposure to the earthquake. In addition, as noted earlier, Yao et al. [37] found that PSOC mediated the association between discrimination and life satisfaction.

#### 3.2.3. Relocation

Eight articles were grouped into the theme of relocation. Relocation was defined as physically moving locations to a new geographic area via a voluntarily move or due to external forces, such as political turmoil, natural disasters, or economic hardship. In this theme, there were two main topics: PSOC aiding in the adjustment to relocation, and the role of culture and life course in understanding PSOC.

Five articles were from the PINE study, which focused on older Chinese immigrants in the U.S. Dong et al. [39] found that increased age, females (compared to males), higher income, having more children, and more years lived in the U.S. were associated with better PSOC. As presented earlier, PSOC was related to social engagement [20,29] and was associated with better health and mental health outcomes [5] but not cognitive functioning [19]. Aside from the PINE studies, Zhang [30] studied PSOC among older women in China who relocated from rural to urban areas. PSOC mediated the relationship between engagement in community activities and relocation adjustment outcomes. Another study explored experiences of Chinese immigrants to New Zealand [22]; here, PSOC was vital to feeling a sense of belonging to multiple communities simultaneously—their new local community and the community from where they emigrated.

Last, a qualitative study explored experiences of PSOC among older women who were forcibly relocated during apartheid in South Africa, including feelings regarding current and former communities [23]. For these women, personal history shaped feelings of PSOC, and there were stronger connections among peers of the same age group and those who shared common interests or beliefs than among generational groups and with current or past physical places.

## 4. Discussion

**Figure 2:**
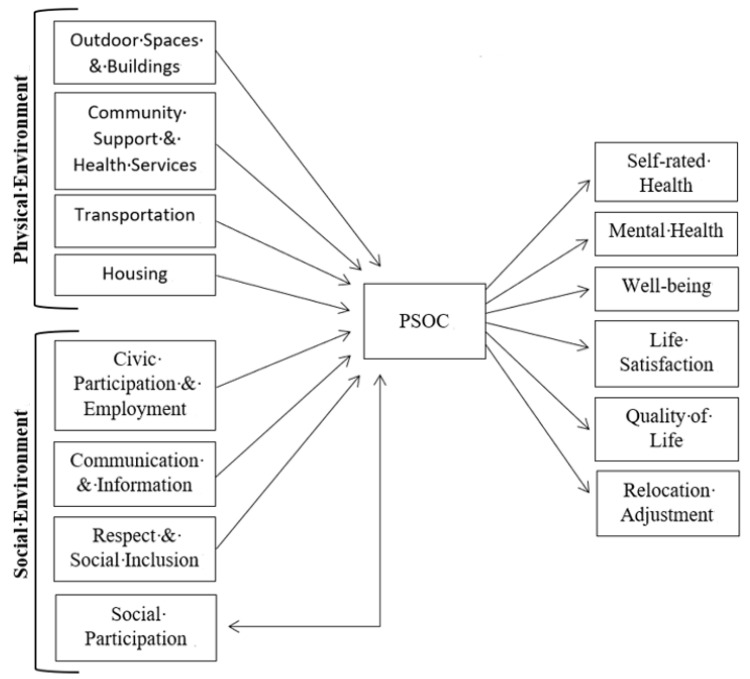
Conceptual Model of Psychological Sense of Community and Related Constructs.

The purpose of this study was to conduct a scoping review on how PSOC has been used in research among community-dwelling older adults and to clarify theoretical propositions by drawing on the Ecological Theory of Aging and the WHO AFC framework. In the final sample of 28 articles, the primary themes were: relevance of PSOC to the AFC framework (both physical and social environments), PSOC as a robust predictor of health and well-being outcomes, and the role of PSOC in relocation. Figure 2 presents an overview of these findings with a conceptual model that shows antecedents and outcomes of PSOC and its role as a mediator.

### 4.1. Physical AFC Environment

Overall, there was a strong relationship between the physical environment and PSOC. Favorable perceptions of one’s neighborhood environment can result in more social inclusion and interaction among community members, whether it be through outdoor spaces or age-friendly transportation options. Neighborhood conditions that are perceived as age-friendly may also increase older adults’ sense of attachment to their community [4]. Further, the physical AFC domains can lead to more fulfillment of needs and influence by offering more features, services, or infrastructure that are conducive to the aging process. These mechanisms provide possible explanations for why an age-friendly physical environment can increase PSOC. Designing age-friendly neighborhoods is a productive strategy to boost PSOC among members.

Age-friendly *Outdoor Spaces and Buildings* provide better accessibility and more opportunities for engagement with others, which can elevate PSOC through increased feelings of membership and emotional connection. Ample public areas are thought to increase connections with others and with their neighborhood, leading to higher PSOC [3]. For example, in Hong Kong, parks are a meeting place and provide opportunities for active and passive social contact, which increases PSOC [6].

The findings of this review also show that the quality of public spaces is important for PSOC [28], and larger amounts of green space may actually inhibit PSOC [2]. For example, while green space is often associated with public parks, it can also represent undeveloped areas where people cannot congregate or spend time, and may increases distances or barriers between residences. Large quantities of public or green space that are not well-maintained may discourage use and result in feelings of disconnection with the community and other members. To enhance PSOC, resources should be used to improve the quality of existing public spaces alongside increasing the quantity and accessibility of spaces. This will increase access and use by older adults and provide more opportunities for engagement in leisure and social activities.

*Community Support and Health Services*, *Transportation*, and *Housing* were also associated with PSOC. Neighborhoods with a higher range and quality of health and community services can result in increased neighborhood satisfaction, opportunities to lead healthier lives, and greater availability of support when needed. The *Community Support and Health Services* domain reflects the PSOC concepts of needs fulfillment and influence. The availability of viable resources and services in the community helps to meet older adults’ needs and provides more control over their environment, such as leveraging services to maintain independence as they age. Housing is a key resource for community-dwelling older adults, and in the AFC framework, it has a focus on maintaining independence, which relates to the needs fulfillment and influence concepts of PSOC. Similar to *Community Support and Health Services*, people residing in areas with less age-friendly housing may feel that their community is not meeting their housing needs and that they have less control over their ability to age in place. Last, available and accessible age-friendly transportation helps older adults maintain connection with others, access services, and feel they are a part of their community. This builds feelings of membership and emotional connection with others, two core concepts of PSOC.

### 4.2. Social AFC Environment

Like the physical environment of AFC, the social environment was relevant to promoting PSOC. The social environment in AFC emphasizes the ability of the community to meet one’s needs, such as a need for social connection and interaction. Connections with other members and a feeling of membership in the community are core concepts of PSOC. Age-friendly social environments increase the likelihood of meaningful interaction among community members and opportunities for bonding, thus boosting PSOC.

Figure 2 depicts a bidirectional relationship between *Social Participation* and PSOC, as more research is needed to clarify the directionality of these or to determine whether they work synergistically. Regardless, there is clearly a strong connection between them. Several studies found that *Social Participation* predicted PSOC [6,7,29,30], while another found that PSOC predicted more social engagement [24]. Additionally, leisure activities such as dog walking [27] or physical activities [32] were relevant to PSOC and may extend beyond the local community. Leisure activities can increase feelings of membership and connection with others, whether it be through increased contact with others or by joining interest-based groups outside of the local neighborhood.

PSOC was also related to *Civic Participation* [33,34,35]. Older adults who are more active in their community may feel greater influence over their environment, which is vital in PSOC. Communities with more opportunities for civic engagement are better able to meet the needs of older adults who are seeking such activities, speaking to the PSOC concept of needs fulfillment. Intervention strategies to promote the civic engagement of older adults, such as options for volunteering or involvement in community improvement, should consider the role of PSOC.

*Communication and Information* was shown to be associated with PSOC, but its impact was not entirely clear in the current literature. While Yu et al. [6] found this domain to be related to better PSOC, Fang et al. [36] found that the increased use of information and communication technology led to worse PSOC. Contrary to the AFC framework, this latter study suggests that the increased use of information and communication technology may lead to more social exclusion and lower PSOC. However, this study focused on PSOC in terms of the local community. It is possible that, while more information and communication technology use may cause one to feel less connected to their local community, they may supplement this loss of connection with meaningful connections in online communities.

The *Respect and Social Inclusion* domain was also found to be related to PSOC [6], and PSOC may in fact protect against feelings of discrimination [37]. The concept of membership in PSOC can explain this relationship. Internalized ageism may lead older adults to feel excluded in their local community, thus resulting in lower levels of PSOC and worse life satisfaction. Conversely, fostering age-inclusive environments presents an opportunity to build PSOC among older adults, and this includes targeting interventions to younger age groups to reduce ageism.

A notable finding in this review was the high prevalence of PSOC as a mediating variable between the aspects of age-friendly environments and health or well-being outcomes; more than one-third of the articles included in this review used PSOC in this way. This pattern in the literature shows that an older adult’s environment, both physical and social, can promote or hinder PSOC, and this, in turn, influences health and well-being outcomes. While not included in the AFC framework, PSOC is an important construct to explain how AFC affects health and well-being outcomes for older adults. Focusing on designing age-friendly communities can enhance PSOC and, ultimately, well-being outcomes. However, as Zhang et al. [3] discuss, neighborhood environments can be difficult to change, so interventions can instead target ways to build PSOC in order to benefit the health and well-being of older adults.

### 4.3. PSOC, Health, and Well-Being

This review shows that PSOC is a robust predictor of better health and well-being outcomes for community-dwelling older adults. This relationship was not surprising, given the theoretical propositions of PSOC, which state that it should be associated with better well-being and mental health outcomes for individuals [11,40].

The somewhat mixed results on physical health were surprising. While some studies found that PSOC predicted better health [5,6], others found non-significant relationships between PSOC and cognitive functioning [19] or physical well-being [25]. However, PSOC is more theoretically aligned with psychological well-being and community involvement outcomes [10], perhaps rather than physical health outcomes, although some studies did show that PSOC promotes better health.

This theme also illustrates the relevance of PSOC to the Ecological Theory of Aging, which stresses the ability of individuals to meet the demands of the environment. PSOC functions as a resource that can be drawn upon to meet the demands of stressors from one’s environment (*environmental press*), whether it be an acute stressor, such as a disaster, or systemic stressors, such as socioeconomic inequity or discrimination. For example, PSOC promotes social relations and connections, which protect against social isolation [38] and lead to positive well-being outcomes.

### 4.4. Relocation and PSOC

Last, research on relocation emphasizes the importance of PSOC to aid in adjustment. The findings from this theme demonstrate how PSOC acts as a resource and protective factor to help meet environmental press that results from immigration or relocation. For example, PSOC can play a pivotal in providing support and helping navigate a new environment [29], such as helping form connections and feelings of belonging in new areas [30].

This theme also highlights the role of culture and life course for understanding PSOC for older adults. As noted by Roos et al. [23], Li et al. [22], and Zhang [30], there was importance of considering the community one left behind along with their current community. Strategies and interventions should focus on building PSOC in current communities, such as targeting those most at risk and promoting social engagement while also helping them maintain a PSOC with their home country or region when applicable.

## 5. Conclusions

This review highlights areas for future research. First, most studies contained samples of Asian older adults. Tang et al. [29] suggest that PSOC is an important construct for Chinese older adults, as it is congruent with cultural values such as collectivism. However, collectivism is an important construct for North American and Western European cultures as well [41]. There remains a need to understand the role of PSOC in diverse cultural and geographic regions. Second, most studies were cross-sectional. This limits causality, especially in structural equational models and mediation analyses, which were commonly used by the articles in this review. Longitudinal designs will clarify directionality and temporality between variables. Third, most antecedents of PSOC were social or physical constructs of the community. While this is a strength of the current state of research, it will be important to identify other predictors of PSOC for older adults, such as developing interventions, the role of natural disasters, and the effects of the COVID-19 pandemic. Fourth, demographic correlates of PSOC should be specifically explored, as PSOC may function differently among groups of older adults. Fifth, future research also needs to consider how “community” is conceptualized. Since most studies used a geographic or neighborhood-level reference for “community,” other types of communities (e.g., online or interest groups) should be included in future research. Finally, a systematic review on this topic is warranted, especially to compare the relative importance and impact of the various domains of AFC on PSOC, as well as which outcomes are most associated with PSOC.

This scoping review had several limitations. First, this review was completed by a single researcher. While this introduces potential bias in selecting and analyzing articles, the author proactively addressed this as thoroughly as possible, including maintaining a detailed journal and consulting with experts in the field. Moreover, sociodemographic correlates of PSOC were not specifically examined; however, some articles reported on these variables, including statistical associations with PSOC. Exploring this topic in more detail may help establish whether there are certain demographic risk factors for lower PSOC among older adults, which Sarason [40] notes is essential to the field.

Overall, this scoping review showed the relevance of PSOC to the physical and social environments and well-being outcomes for community-dwelling older adults. The results are relevant for refining theory and research on PSOC and for guiding policy and practice. A more robust understanding of PSOC will help further knowledge about how AFC is related to well-being outcomes, as PSOC provides a link between an older adult’s environment and outcomes. This was especially strong for topics such as life satisfaction, well-being, health, and quality of life. Finally, this research supports the relevance of PSOC in designing cities and communities that are age-friendly. Practitioners, planners, and policy makers can improve the health and well-being of older adults by focusing on strategies and interventions that enhance their PSOC.

## Data Availability

Not applicable.

## Supplementary Materials

The following are available online at www.mdpi.com/article/10.3390/ijerph19148395/s1, Table S1: Preferred Reporting Items for Systematic Reviews and Meta-Analyses Extension for Scoping Reviews (PRISMA-ScR) Checklist.
